# Defining Driver DNA Methylation Changes in Human Cancer

**DOI:** 10.3390/ijms19041166

**Published:** 2018-04-12

**Authors:** Gerd P. Pfeifer

**Affiliations:** Center for Epigenetics, Van Andel Research Institute, 333 Bostwick Avenue NE, Grand Rapids, MI 49503, USA; gerd.pfeifer@vai.org

**Keywords:** DNA methylation, hallmarks of cancer, 5-methylcytosine, cell differentiation, genomic instability

## Abstract

Human malignant tumors are characterized by pervasive changes in the patterns of DNA methylation. These changes include a globally hypomethylated tumor cell genome and the focal hypermethylation of numerous 5′-cytosine-phosphate-guanine-3′ (CpG) islands, many of them associated with gene promoters. It has been challenging to link specific DNA methylation changes with tumorigenesis in a cause-and-effect relationship. Some evidence suggests that cancer-associated DNA hypomethylation may increase genomic instability. Promoter hypermethylation events can lead to silencing of genes functioning in pathways reflecting hallmarks of cancer, including DNA repair, cell cycle regulation, promotion of apoptosis or control of key tumor-relevant signaling networks. A convincing argument for a tumor-driving role of DNA methylation can be made when the same genes are also frequently mutated in cancer. Many of the most commonly hypermethylated genes encode developmental transcription factors, the methylation of which may lead to permanent gene silencing. Inactivation of such genes will deprive the cells in which the tumor may initiate from the option of undergoing or maintaining lineage differentiation and will lock them into a perpetuated stem cell-like state thus providing an additional window for cell transformation.

## 1. Introduction

DNA methylation involves the addition of a methyl group to position 5 of the DNA cytosine ring by DNA methyltransferase enzymes. This modification occurs predominantly at CpG dinucleotide sequences in mammals. DNA methylation patterns are established and maintained by a small family of DNA cytosine-5 methyltransferase enzymes (DNMT1, DNMT3A, DNMT3B), which transfer methyl groups from the co-factor *S*-adenosyl-l-methionine to DNA cytosine bases predominantly within the CpG dinucleotide sequence context [1]. The level of methylation at individual CpG sites as well as the distribution of methylated and unmethylated CpGs across the genome is linked to developmental state and cellular differentiation processes in mammalian organisms [2]. Two waves of major CpG methylation remodeling consisting of genome-wide DNA demethylation and then remethylation occur during the earliest stages of embryogenesis and in primordial germ cells [3]. More subtle alterations of methylation patterns accompany the development of specific cell lineages and tissue formation. 

The first observations of aberrant DNA methylation in cancer tissues were reported almost 40 years ago when global methylation analysis by chromatographic techniques indicated a reduction of DNA methylation levels in several types of malignancies in comparison with the corresponding normal tissue samples from which the tumors were derived [4,5,6]. These changes were confirmed at the level of specific genes or at repetitive sequences using Southern blotting techniques [7,8]. 

**Figure 1:**
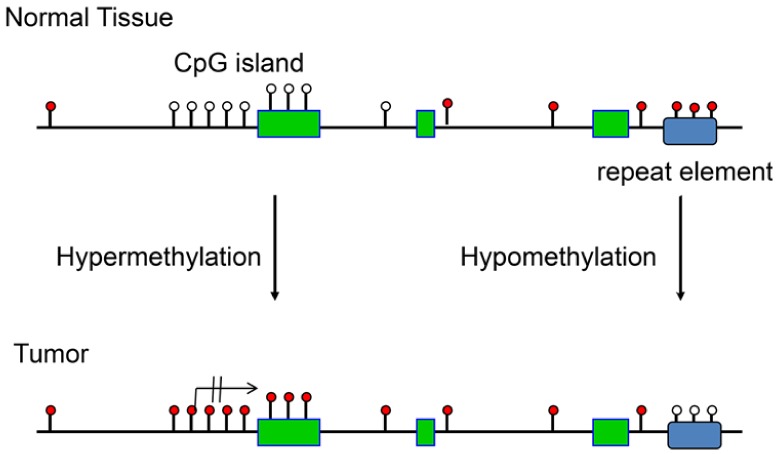
Schematic outline of the most relevant DNA methylation changes observed in human cancers. These events include CpG-island-specific DNA hypermethylation often occurring at gene promoters, which locks the affected gene into an inactive state. Loss of DNA methylation (hypomethylation) occurs genome-wide and is often observed at repetitive regions of the genome. White circles indicate unmethylated CpG sites and red circles show methylated CpG sites. The crossed-out arrow indicates the transcription start site and the permanent lack of transcription after DNA methylation. Green boxes show exons and the blue rectangle marks the position of a repetitive element.

Besides global loss of 5-methylcytosine (5mC), the other prevalent phenomenon occurring in cancer is the hypermethylation of CpG islands (Figure 1), which was initially reported for the calcitonin gene in the mid-1980s [9]. CpG islands are characteristic landmarks of vertebrate genomes with much higher than average frequencies of the CpG dinucleotide sequence [10]. In vertebrates, this dinucleotide is strongly depleted due to mutational processes ascribed to the modified base, which results in CpG frequencies of only about 1/5 of the expected one [11,12]. However, within CpG islands, CpGs remain unmethylated and are not depleted, and thus they appear about 5 times more frequent than they are in the rest of the genome. Specific protection mechanisms very likely exist that result in exclusion of DNA methylation from CpG islands or in its removal by Ten-Eleven-Translocation (TET) protein-catalyzed 5mC oxidation in situations when methylation has been deposited erroneously into such sequences by DNMT enzymes. In many, if not all types of human cancer, these methylation protection mechanisms appear to be insufficient resulting in the phenomenon of CpG island hypermethylation. In most cancers, several hundred CpG islands are known to undergo methylation in most individual tumor specimens [13,14]. Some specific types of tumors, for example low-grade gliomas and certain colorectal cancers, are characterized by a so-called CpG island methylator phenotype (or CIMP), a condition in which a few thousand CpG islands become methylated simultaneously in an individual cancer sample [15,16].

The mechanisms of how these methylation changes in cancer occur are only partially understood. Different genomic regions, such as late-replicating or repetitive regions seem to undergo DNA hypomethylation in a preferential manner [17,18], whereas CpG islands associated with a certain chromatin configuration reflected by the Polycomb mark (histone H3 lysine 27 methylation) are highly susceptible to DNA hypermethylation [19,20,21,22,23]. It is also possible that genetic mutations affecting chromatin regulatory processes, such as in TET genes or their associated co-factor pathways (isocitrate dehydrogenase genes), will favor the state of CpG island hypermethylation [24].

Although we now have a rather complete understanding of the nature of DNA methylation changes in malignant tissue due to application of genome-scale methylation mapping techniques, it has been much more difficult to pinpoint those methylation changes that have a functional role in tumorigenesis as opposed to being perhaps only a consequence of the malignant state. In a way, this situation is reminiscent of the challenge that exists for distinguishing driver mutations in cancer from innocuous passenger mutations. A tumor genome may harbor tens of thousands of somatic mutations and yet only a handful of them may be critical for cancer formation. Similarly, a tumor epigenome may contain a thousand hypermethylated CpG islands but only very few may be relevant for cell transformation. One other aspect to consider is the possibility that methylation changes are secondary to other events that have occurred at the chromatin level; for instance, a change in gene expression may occur in tumors as the result of acquisition or loss of a certain transcription factor, and this event may then trigger the methylation change. This argument is based on the now widely accepted idea that DNA methylation is the default state and that changes of genome occupancy of various DNA binding proteins can trigger methylation changes [25,26]. For these reasons, it has been a major challenge to identify true “driver methylation” events and to distinguish them from “passenger methylation” events [27]. A driver methylation event can be defined as a methylation event that results in inactivation of a particular gene, leading to phenotypic consequences that promote cell transformation or advance the malignant progression of a cell. A passenger methylation event is the methylation of a gene or a set of genes which occurs without having any noticeable effect on tumor progression.

## 2. DNA Hypomethylation during Tumor Formation

**Figure 2:**
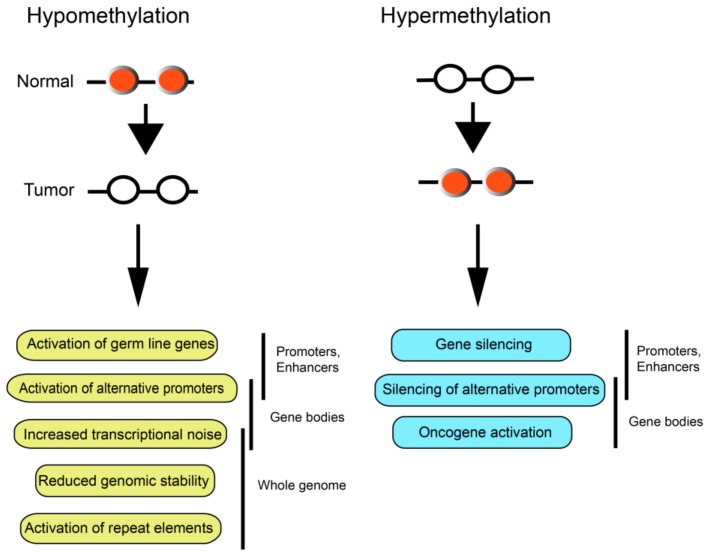
Genome-scale consequences of DNA hypomethylation and DNA hypermethylation in cancer. DNA hypomethylation can lead to perturbations in gene expression or genomic instability. Likewise, DNA hypermethylation may promote widespread changes in gene expression patterns through different mechanisms. White circles indicate unmethylated CpG sites and red circles show methylated CpG sites.

In most mammalian tissues, approximately 3% to 5% of all cytosines are methylated. During global DNA hypomethylation of tumor genomes, there is a minor to moderate reduction of the number of methylated cytosines amounting to a loss of about 5% to 20% on average of the 5mC bases. Because this loss occurs so globally, it has been difficult to assign this phenomenon to specific molecular pathways or genes that are affected by the loss of methylation (Figure 2). 

One line of reasoning has been that the methylation loss will be accompanied by a less compact chromatin structure leading to the possibility of genomic instability. For example, mice deficient in *Dnmt1* have increased tumor burden, which may be ascribed to loss of heterozygosity events and reduced genomic stability [28,29].

Repetitive genomic regions, which account for about 55% of the human genome, are particularly susceptible to 5mC loss. These sequences include long interspersed nuclear elements (LINE) and Alu-type repetitive regions, two major types of interspersed repeat elements, as well as various classes of endogenous retroviruses (for example, human endogenous retrovirus K; HERV-K) and centromeric satellite repeats. Due to their abundance, methylation of LINE-1 or *Arthrobacter luteus* (Alu) repeats has often been used as surrogate markers for global DNA methylation levels [30]. Since DNA methylation and repressive chromatin marks in the form of histone H3 lysine 9 (H3K9) methylation often cooperate to silence repetitive genomic regions, the loss of DNA methylation is likely one way to permit activation of gene expression at repetitive regions. 

LINE-1 activation may indeed be a component of carcinogenesis. Sequencing studies of cancer genomes from several hundred patients revealed that tumors from a substantial fraction of the patients had somatic retro-transpositions of LINE-1 elements [31]. The activity of individual LINE-1 elements was correlated with LINE-1 promoter hypomethylation events. Some retro-transpositions occurred in known cancer genes. Such events are expected to influence tumorigenesis by changing the structure of the genome through insertional mutagenesis.

One other phenomenon associated with the cancer epigenome is the large-scale rearrangement of the three-dimensional chromatin architecture. There are rearrangements to large domains of chromatin blocks (e.g., those regions marked by H3K9me2) and to the nature of lamina-associated domains in cancer [18,32]. The lamina-associated domains are preferentially hypomethylated. These chromosomal re-localization and chromatin structural transitions may favor inappropriate chromosomal breakage and rearrangements. However, it is difficult to determine if DNA hypomethylation is initially causing these structural perturbations or if hypomethylation follows the re-organization of chromatin structure. Earlier studies with hypomorphic mouse mutants of *Dnmt* genes have suggested that methylation loss is potentially causative for cancer formation [28,29]. However, studies of human early tumorous lesions, such as benign tumors, have not resulted in a clear picture as to the timing of global DNA hypomethylation during tumor progression.

Gene-specific DNA hypomethylation events might also contribute to cancer. There is a group of genes that becomes activated in tumors by loss of DNA methylation at the promoter regions [33,34]. These genes fall into the category of germ line-specific genes and are also referred to as “cancer testis genes” or “cancer germ line genes” since many of them are active only during spermatogenesis. These germ line expressed genes are normally silenced in most somatic tissue by DNA methylation. Initially, these genes were discovered in melanoma tumors as antigens recognized by cytotoxic T lymphocytes and some of them were give the name *MAGE* (melanoma antigen gene). These genes were expressed also in many other types of cancer but not in normal somatic tissues revealing them as suitable biomarkers of malignancy and even as potential therapeutic targets due to their unique cancer-specific expression patterns. In total, there are over 250 genes characterized as cancer testis genes with many of them being localized on the X chromosome. One major branch of these genes is the MAGE family, which is evolutionary conserved and consists of over 50 family members. These genes encode ubiquitin ligases that function during germ cell development in reproductive organs. Although the cancer testis genes are markers of malignancy, their functional involvement in carcinogenesis has been less clear. Such a role would best be proven if they would have an oncogenic role in somatic tissues when aberrantly expressed [34]. Indeed, overexpression and depletion experiments have shown that several cancer testis antigen genes have a pro-proliferative effect. For example, the genes Maelstrom (*MAEL*) [35] and Piwi-like 1 (*PIWIL1*) have a positive effect on phosphoinositide-3 (PI3) kinase/AKT signaling and cell proliferation pathways [36]. Some of the MAGE proteins were shown to bind to and incapacitate well known tumor suppressor proteins such as tumor suppressor protein 53 (TP53) and Retinoblastoma (RB) by different mechanisms [37]. One interesting member of the cancer testis gene family is *BORIS*/*CTCFL*, which encodes a homologue of the insulator protein CCCTC binding factor (CTCF). *BORIS*/*CTCFL* was shown to be upregulated in testicular and ovarian cancer where the encoded protein leads to upregulation of the telomerase reverse transcriptase (hTERT) gene promoting cell immortalization [38]. Other members of the activated germ line genes were shown to participate in metabolic disturbances and in promoting genomic instability [34]. Even though each one of these genes may have only a moderate impact on tumor progression by itself, their combined activation likely will provide a more relevant tumor-driving role, which in its totality may overcome the properties of these gene products as potential antigens favoring tumor rejection.

One additional outcome of genome-wide DNA hypomethylation is its likely effect on global gene transcription patterns. On the genome scale, the expression level of genes is positively correlated with the level of methylation within the transcribed regions, also referred to as gene bodies [39]. This correlation was first demonstrated for genes on the active X chromosome [40] but applies to all chromosomes of the human genome [39]. The functional role of intragenic DNA methylation in promoting gene expression is still unclear, but recent findings suggest that a loss of gene body DNA methylation will increase transcriptional noise by allowing the unscheduled activation of intragenic transcripts that could, for example interfere with sense strand transcription [41]. Therefore, it is conceivable that a reduction of gene body methylation in cancer will lead to a global perturbation of transcriptional fidelity, which in itself might be a contributing factor for the malignant cell state. Furthermore, gene body DNA methylation may control alternative promoters of genes [39,42]. In this case, DNA hypomethylation may activate alternative transcripts of genes that are otherwise suppressed by intragenic DNA methylation.

## 3. Tumor-Driving DNA Hypermethylation Events

Genome-wide hypermethylation of CpG islands is observed not only in most primary and metastatic tumors [14] but is already seen in premalignant lesions, for example in aberrant crypt foci of the colon [43] or in actinic keratosis lesions of the skin [44]. A tumor-driving role of a gene undergoing hypermethylation in cancer is best rationalized when the methylation event affects regulatory gene sequences such as enhancer or promoter regions. In these cases, DNA methylation is generally associated with gene silencing. On the other hand, hypermethylation of CpG-rich regions within gene body regions can be associated with at least two possible outcomes. The first is the silencing of one of two or more alternative promoters of a gene leading to a change in expression of specific transcript isoforms [39,42]. Gene body hypermethylation also is associated with higher gene expression levels at least at a global genome scale. If this phenomenon occurs in genes harboring oncogenic properties, the gene body hypermethylation may promote carcinogenesis by oncogene activation [45] (Figure 2). More commonly, however, and when affecting promoters, CpG island hypermethylation will lead to gene silencing. 

**Figure 3:**
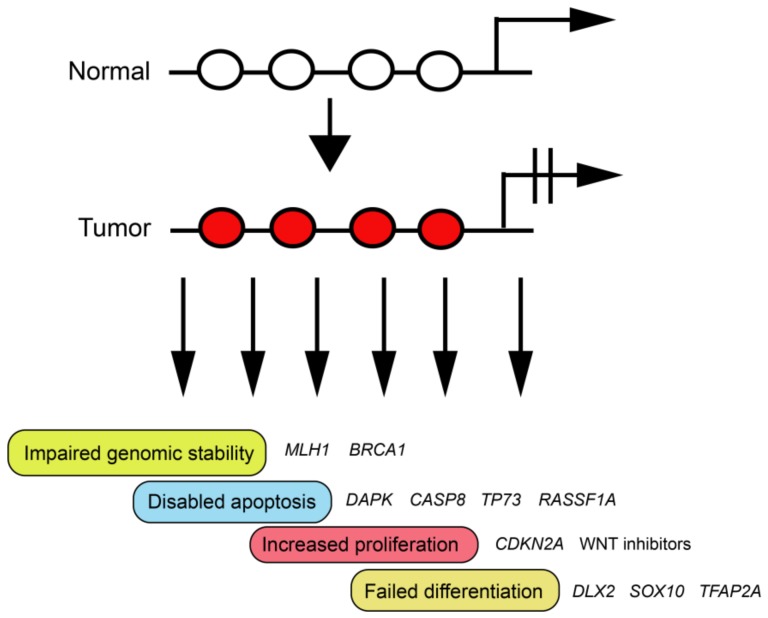
Potential tumor-driving consequences of CpG island hypermethylation. When DNA hypermethylation occurs at gene regulatory elements, the resulting gene silencing may have a tumor-promoting effect if the targeted genes function within the framework of the ‘hallmarks’ of cancer. These genes include, for example, DNA repair genes, genes encoding factors involved in cell growth control, or genes involved in promoting apoptosis or cell differentiation. Some examples are shown. White circles indicate unmethylated CpG sites and red circles show methylated CpG sites.

A tumor-promoting effect of methylation-induced silencing events can be expected if the affected genes participate in functional pathways described as the “hallmarks of cancer” (Figure 3). Those include the control of cell proliferation, induction of apoptosis or senescence, angiogenesis, cell adhesion, invasion and metastasis, DNA repair and genomic stability, anti-inflammatory responses, and a few other possible mechanisms [46].

## 4. Classical Tumor Suppressor Genes (TSGs)—*BRCA1*, *MLH1*, *APC*, and *CDKN2A*

The following examples illustrate that DNA hypermethylation of known tumor suppressor genes does occur as an alternative to mutational inactivation or gene copy loss and thus is very likely representing a true tumor-driving event. 

The discovery of cancer-associated hypermethylation of the gene cyclin-dependent kinase inhibitor 2A (*CDKN2A*), coding for a CDK inhibitor protein also known as p16, marked one of the earliest demonstrations of a tumor-driving role of DNA hypermethylation [47]. Inhibition of cell cycle promoting kinases is an important proliferation control mechanism and the inactivation of this mechanism is expected to lead to enhanced cell growth. Methylation of *CDKN2A* occurs in many types of malignancy including breast cancer, head and neck cancers, gliomas, and melanomas. Importantly, *CDKN2A* can be inactivated by several mutually exclusive events including homozygous loss, base substitution mutations, and by promoter methylation. 

The breast cancer susceptibility gene breast cancer 1 (*BRCA1*), which is mutated in hereditary breast and ovarian cancers, contains aberrant promoter methylation in a smaller subset of sporadic breast and ovarian tumors [48,49]. This methylation correlates with a loss or reduction of BRCA1 protein. The BRCA1 protein functions in DNA repair by promoting homologous recombination and is essential for maintaining genome integrity. This data indicates that methylation-induced silencing of *BRCA1* can contribute to defects in homology-directed DNA repair in the affected individuals. In contrast to *BRCA1*, methylation-mediated silencing of *BRCA2* is relatively rare in breast or ovarian cancers.

Mutations in DNA mismatch repair genes have been found in familial cancer predisposition syndromes and in sporadic tumors, notably colorectal tumors with microsatellite instability. However, a majority of mismatch repair deficient sporadic colorectal tumors do not contain mutations in any of the DNA mismatch repair genes such as *MSH2*, *MLH1*, *MSH6* or *PMS2*. Instead, these tumors commonly present with DNA hypermethylation of the *MLH1* gene promoter [50]. Generally, this promoter methylation does not occur or is less prevalent in tumors that also carry mutations in mismatch repair genes. Methylation can lead to biallelic inactivation of *MLH1* and to loss of protein expression. This inactivation can be reversed by treatment with the DNA methylation inhibitor 5-aza-deoxycytidine [51]. Since *MLH1* promoter methylation is a loss of function event analogous to gene mutation, this represents a convincing example for a driver methylation event in tumorigenesis.

Germline mutations of the tumor suppressor gene adenomatous polyposis coli (*APC*) are associated with the familial cancer syndrome adenomatous polyposis coli, which predisposes its carriers to early onset colorectal cancer. APC is a negative regulator of the Wingless/Int (WNT) signaling pathway. Methylation of the promoter region of the *APC* gene leads to gene silencing and may contribute to carcinogenesis [52]. *APC* methylation is not only found in colorectal tumors but also in esophageal cancer, gastric cancer, pancreatic and prostate cancers as well as in some other tumor types. 

## 5. Other Likely Tumor Suppressor Genes: DNA Repair Genes, Proapoptotic Genes, and Anti-Proliferative Factors Functioning in Signaling Pathways

The inactivation of DNA repair function will likely increase the incidence of mutations, both at the single base level or at the chromosomal level depending on which repair pathways have been disabled. Several DNA repair processes are compromised in tumors, most notably by germ lines mutations. Examples include the defects in nucleotide excision repair because of mutations in the Xeroderma pigmentosum gene group (e.g., *XPA*, *XPC*, *XPF*). Mutations in DNA mismatch repair genes, as discussed above, lead to a hypermutator phenotype often manifesting itself as microsatellite instability. Genetic defects in base excision repair genes are more rarely associated with cancer. DNA double strand break repair and recombination repair pathways are compromised by mutations in *BRCA1*, *BRCA2*, *RAD51* paralogs, and a few other genes. Several alternative epigenetic pathways for DNA repair gene inactivation have already been discussed above, including *MLH1* and *BRCA1* methylation. However, cancer-associated methylation of the many other DNA repair genes is a relatively rare occurrence in human cancer [53]. One notable exception is the direct DNA damage reversal enzyme MGMT (O6-methylguanine methyltransferase), a protein which transfers methyl groups from the mutagenic DNA base O6-methylguanine onto the protein itself leading to its own inactivation. Guanine-O6 methylation leads to the formation of a methylated base with disrupted base pairing capacity where the O6-methylguanine pairs with thymine rather than cytosine during DNA replication thus promoting G:C to A:T mutations. The *MGMT* gene is epigenetically inactivated by DNA methylation in colorectal cancers, gastric cancers, head and neck squamous cell carcinomas, non-small cell lung cancers and most prominently in gliomas [54]. Methylation silencing of *MGMT* is expected to diminish the efficiency of O6-alkylguanine repair. It was shown that glioma patients with a methylated *MGMT* gene had a better survival rate when treated with the alkylating agent temozolomide or other alkylating agents when compared to patients with an unmethylated promoter presumably because of increased cell killing by the chemotherapy agent [55]. 

Evasion of apoptosis is a hallmark of cancer. One would expect that DNA methylation events that diminish apoptosis are potential tumor-driving events. Indeed, methylation silencing of several proapoptotic genes has been reported in malignant tumors. One of the first examples of methylation of a pro-apoptotic gene was death-associated protein kinase (*DAPK*), which occurs in B cell malignancies and many other cancer types [56,57]. Similarly, methylation of the caspase 8 gene (*CASP8*), which encodes a cysteine protease regulated in a death-receptor-dependent and independent manner, was found in neuroblastomas and other tumors [58]. Another protein with apoptosis-inducing function is TP73, a paralogue of the well-known tumor suppressor TP53. Methylation of the *TP73* promoter is found in neuroblastomas, melanomas, and several other cancers [59,60]. 

The constraint of proliferation-promoting signaling pathways is an important aspect of growth control mechanisms. One of these pathways elicited by the cyclin-dependent kinase inhibitors, which exert control over the cell cycle, has already been discussed by highlighting the common methylation-related silencing of the *CDKN2A* gene. A related gene, *CDKN2B*, is located next to the *CDKN2A* locus, end encodes the cyclin-dependent kinase inhibitor protein p15. While both genes can be simultaneously deleted in tumors, methylation of *CDKN2B* is also found in various cancers [61]. Furthermore, an alternative splicing process with different promoter usage at the *CDKN2A* locus generates the transcript *p14ARF*, which encodes a protein which antagonizes MDM2-dependent TP53 degradation. Methylation of the *p14ARF* gene also is seen commonly in many tumor types. This silencing event is expected to disable the TP53 tumor suppressor function. 

The WNT pathway is a growth-promoting module with particular relevance for intestinal stem cells and tumors arising from them. There are several inhibitory molecules that can control WNT pathway activity. One of them is APC, which has been discussed earlier. Other WNT inhibitory molecules are the signaling antagonists secreted frizzled-related proteins (SFRPs). Methylation-induced silencing of *SFRP1* and other members of this family has been found in colorectal cancer, but also in breast cancers, hepatocellular carcinomas, and other cancers [62]. Other methylated genes for different types of other WNT inhibitors include *DKK1*, *WIF1*, *WNT7A*, and several others. 

One other example in which methylation of signaling network control elements may promote cancer formation is the Hippo tumor suppressor pathway. First studied in *Drosophila*, the Hippo pathway restrains cell proliferation and functions in organ size control [63]. The pathway includes a central kinase cassette containing the Hippo kinase or its mammalian counterparts, the mammalian sterile 20-like kinases 1 and 2 (MST1 and MST2). After phosphorylating and activating a downstream kinase, large tumor suppressor (LATS), the phosphorylation events culminate in inhibitory phosphorylation and cytoplasmic sequestration of the oncogenic protein Yes-associated protein (YAP), a transcriptional co-activator and pro-proliferative component of the pathway. Deletion of the *MST1*/*MST2* genes or overexpression of *YAP* produces liver cancer in mice [64]. Somewhat unexpectedly, this pathway is rarely mutated in human cancer [63]. Instead, methylation of the *MST1* and *MST2* promoters has been found in soft tissue sarcomas [65]. A few positive regulators of MST kinases have been identified including the Ras association domain family (RASSF) proteins [66]. One RASSF family member (RASSF1A) is encoded by one of the two major splice variants of the *RASSF1A* gene [67]. Methylation of the *RASSF1A* gene is one of the most frequent methylation events in human cancer [68]. Methylation of this gene is found in almost every type of human cancer and is commonly already detectable in early preneoplastic lesions. RASSF1A positively regulates the Hippo growth control pathway including its pro-apoptotic output through the MST1/2 kinases [69]. The RASSF proteins contain a RAS association domain that allows signaling through activated RAS oncogenic proteins. Therefore, in addition to their role in the Hippo pathway, the RASSF proteins may also function as negative regulators of RAS signaling [70].

## 6. Transcription Factors Involved in Differentiation Pathways

With the advent of genome-scale DNA methylation mapping techniques, the field moved from a specific gene-centered approach to cataloguing the entirety of DNA methylation changes found in tumors. The various methylation mapping techniques were based initially on restriction enzymes that are blocked by CpG methylation, but soon included methods that employ antibodies against 5mC or proteins that bind to 5mC with high selectivity specifically to enrich methylated gene loci. Other methods that provide the most comprehensive overview use whole genome bisulfite sequencing. More limited scale bisulfite-based approaches such as the Illumina array platforms have also very commonly been used. These genome-scale studies revealed that the most frequently methylated genes in cancer cells were genes encoding developmental transcription factors [20]. One class of these genes are the *HOX* genes and other homeobox gene family members. For example, the genes *HOXA7* and *HOXA9* are commonly methylated in lung tumors and other cancers [21]. The homeobox genes *OTX1* and *NR2E1*/*TLX1* are methylated in 100% of the lung squamous cell carcinomas tested [17]. These transcription factor genes are best known for their roles in early developmental processes and in the differentiation of specific cell lineages or organs. In adult tissues, they are generally expressed at very low levels and are occupied by repressive Polycomb chromatin complexes which catalyze the formation of the histone mark histone H3 lysine 27 trimethylation (H3K27me3). The same complexes also mark these genes, often in a bivalent state with active (H3K4me3) and inactive marks (H3K27me3), in embryonic stem cells where they are poised for expression upon cell lineage differentiation [71]. Because of their low expression state in normal tissues, it has been difficult to rationalize the methylation of Polycomb target genes as a tumor driving event. However, some of these tumor-methylated genes have a role in differentiation within the same tissue where the corresponding tumor contains these genes in a methylated state. This finding was initially reported for astrocytomas, where several genes important for neuronal differentiation (for example *PAX6*, *DLX2*, and *SIM1*) are hypermethylated in the tumors [72]. Since DNA methylation imposes a probably more stable, and more long-term silencing on the affected genes, it is likely that inactivation of these differentiation factors is incompatible with the differentiated state of the cells in which the methylation occurs. When tumor-initiating cells are affected by the methylation event, their failure of undergoing or sustaining normal differentiation may contribute to their propensity to become transformed by additional oncogenic events. Another example is malignant melanoma, in which genes encoding the melanocyte lineage differentiation factors SRY-related HMG box 10 (SOX10), microphtalmia-associated transcription factor (MITF), and transcription factor AP2 alpha (TFAP2A) are commonly affected by promoter methylation [59]. In conclusion, methylation and stable silencing of genes encoding many developmental transcription factors is a common event in cancer. Although a functional role in tumor formation has not been explicitly proven for these types of DNA methylation events, a theoretical framework for their functional significance is now apparent. 

## 7. Conclusions

Genome-wide DNA methylation analysis has provided comprehensive overviews of the scale of tumor-associated DNA methylation changes. There are at least two major challenges remaining. First, it is not at all clear how these DNA methylation changes are initiated. Second, and as discussed in this article, we still need to understand which of the numerous DNA methylation changes are truly tumor-driving events. Although changes in DNA methylation, in the form of both overall DNA hypomethylation and localized DNA hypermethylation at CpG-rich promoters have been known for several decades, it is still a challenge to pinpoint specific methylation changes and their associated mechanisms as tumor-driving processes. The mechanisms of how DNA hypomethylation promotes tumorigenesis in human cells are particularly unclear. Although several examples now exist for a tumor-driving role of methylation-induced gene silencing, the physiological effects of the clear majority of de novo DNA methylation events are not known. One way to investigate the potential of a methylated gene in tumor formation is to delete the gene, either in cultured normal cells or in mice and then observe effects of this deletion on the tumorigenic phenotype of the manipulated cells or animals. More relevant would be the specific methylation of the gene of interested using epigenetic editing techniques. These techniques are now being developed and include, for example, the CRISPR/dCas9-mediated introduction or removal of methyl groups from targeted gene loci. However, some of these techniques still lack the desired specificity and induce gene methylation at many non-targeted loci. Improvement of these approaches, perhaps in combination with multiplexed, library-type screening systems should allow a more precise dissection of the roles of many of the observed methylation changes as they occur in human cancer.

There are now many examples clearly suggesting a tumor-promoting role of hypermethylation at specific gene promoters. These examples include the methylation of genes controlling DNA repair and genomic stability. Genes relevant for induction of apoptosis, for controlling pro- and anti-proliferative signaling networks, and genes encoding transcription factors involved in lineage differentiation are also subject of tumor-specific DNA methylation. Individual tumors often will have multiple genes from these pathways in a hypermethylated state suggesting that the sum of these methylation events will provide an even stronger tumor-promoting force than DNA methylation occurring at single genes. 

## Abbreviations

5mC	5-methylcytosine
APC	adenomatous polyposis gene
BRCA1	breast cancer 1 gene
CDKN2A	cyclin-dependent kinase inhibitor 2A gene
DAPK	death associated protein kinase
DNMT	DNA methyltransferase enzyme
LINE	long interspersed nuclear element
MAGE	melanoma antigen gene
MGMT	O6-methylguanine methyltransferase
MLH1	MutL homologue 1
RASSF1A	ras association family 1A
SFRP	secreted frizzled-related protein
TET	Ten-Eleven-Translocation enzyme
